# Vibrotactile spatial acuity on the back

**DOI:** 10.1177/03010066241258969

**Published:** 2024-06-11

**Authors:** Myrthe A. Plaisier, Cahelle S.J.M. Vleeshouwers, Nynke Boonstra, Yueying Shi, Sam J.I. van der Velden, Wouter K. Vos, Astrid M.L. Kappers

**Affiliations:** Human Technology Interaction, Eindhoven University of Technology, Eindhoven, The Netherlands; Human Technology Interaction, Eindhoven University of Technology, Eindhoven, The Netherlands; Human Technology Interaction, Eindhoven University of Technology, Eindhoven, The Netherlands; Human Technology Interaction, Eindhoven University of Technology, Eindhoven, The Netherlands; Human Technology Interaction, Eindhoven University of Technology, Eindhoven, The Netherlands; Elitac Wearables B.V., Utrecht, The Netherlands; Dynamics and Control, Control Systems Technology, and Human Technology Interaction, Eindhoven University of Technology, Eindhoven, The Netherlands

**Keywords:** vibrotactile perception, horizontal and vertical spatial acuity, wearables

## Abstract

Vibrotactile feedback can be built into clothing such as vests. This means that often vibrotactile information is presented to the back. It is known that the back has a relatively low spatial acuity. Spatial acuity varies across different limbs and sometimes with different locations on a limb. These known anisotropies suggest that there might be systematic variations in vibrotactile spatial acuity for different areas of the back and also for different orientations (i.e. horizontal vs. vertical). Here we systematically measured spatial acuity in four areas of the back for both horizontal and vertical orientations. The results show no significant differences in spatial acuity for the back areas that were tested. Spatial acuity was, however, higher in the horizontal direction than in the vertical direction by roughly a factor of two. This means that when designing vibrotactile displays for the back the tactor density can be lower in the vertical direction than in the horizontal direction and density should be constant for different areas of the back.

Wearable haptic displays are gaining traction and haptic technology is often built into clothing. While many types of haptic actuators exist, often vibration motors are incorporated in practice. When these are built into a belt, vest, or backpack, vibrotactile feedback can be provided to the torso. These can be used to provide, for instance, cues for navigation (e.g. Ertan et al., 1998; Srikulwong & O’Neill, 2013; van Erp et al., 2017). They can also be used to convey shapes and letters that can be recognised by the wearer (Loomis, 1974; Wu et al., 2011, 2012). Due to practical reasons, often the back is chosen as the body part to display the tactile information on. Advantages are the large surface area of the back and that it is relatively stationary (Hoffmann et al., 2018). Furthermore, it is easy to put a vest on making it easy to use in daily life. The main disadvantage of presenting vibrotactile information on the back area is that the back has a relatively low spatial acuity (Weinstein, 1968; Weber et al., 1996). Furthermore, distance perception (Plaisier et al., 2020; Nicula & Longo, 2021) as well as direction perception (Kappers et al., 2020; Plaisier & Kappers, 2022) are anisotropic. Such perceptual effects need to be accounted for when designing vibrotactile displays. For optimising vibrotactile displays that are worn on the back, it is important to gain insight into perceptual performance for vibrotactile stimulation in general and vibrotactile spatial acuity specifically. One of the easiest ways to reduce the costs of a vibrotactile wearable is by reducing the number of actuators and thus actuator density. The vibrotactile spatial acuity provides a lower threshold for actuator spacing. Spacing actuators closer together than the spatial acuity will usually not lead to better perceptual performance. If there are large variations in spatial acuity over different areas of the back or for different orientations (i.e. horizontal or vertical) manufacturers could consider using an anisotropic actuator density to reduce the number of actuators without compromising spatial acuity.

Spatial acuity on the skin in general is known to vary over body areas. For pressure stimuli, it has been found that spatial acuity varies over body regions and increases for more distal areas (Weinstein, 1968). Furthermore, Weber found that spatial acuity of pressure stimuli is often higher across the body width than along the body length (Weber et al., 1996). There are some indications that vibrotactile spatial acuity varies for different body areas. For instance, vibrotactile spatial acuity was found to be higher on the body midline near the navel and on the spine (van Erp, 2005). Another study, however, found lower accuracy of spatial localisation of vibration in the region around the spine compared to peripheral regions for stimuli in the horizontal direction, but no difference for stimuli in the vertical direction (Hoffmann et al., 2018). In that same study, it was also found that the localisation accuracy of vibrotactile stimuli was higher in the horizontal direction than in the vertical direction (Hoffmann et al., 2018). Van Erp did not find a difference in spatial acuity for vertical versus horizontal presentation of the vibrotactile stimuli on the torso (van Erp, 2005).

When measuring vibrotactile spatial acuity the result may depend on the specific area of the back, the specific actuator that is used, whether stimulation is sequential or simultaneous and on which psychophysical method is used. Various studies have therefore yielded different discrimination thresholds for the vibrotactile two-point threshold and reported thresholds to range from 13 to 60 cm (van Erp, 2005; Hoffmann et al., 2018; Eskildsen et al., 1969; Stronks et al., 2016; Jóhannesson et al., 2017; Špakov et al., 2015). To be able to compare measured spatial acuity across different areas of the back as well as orientation it is necessary to vary these factors while keeping all other variables constant.

In the current study, we systematically mapped the vibrotactile spatial acuity for different areas on the back. These areas differed in height on the back and in whether they were on the left or right side of the spine. Spatial acuity was measured in two orientations: horizontal (i.e. across the width of the back) and vertical (i.e. along the length of the back). We used a commercially available tactor string for this experiment which incorporated a very commonly used type of vibration motor. As a measure of spatial acuity, we determined the smallest tactor spacing for which participants could identify the direction (up-down or left-right) of two sequentially vibrating vibration motors. We chose this measure because vibrotactile displays are often used to display shapes that are traced by switching vibration motors on and off sequentially (e.g. Yanagida et al., 2004; Loomis, 1974; Wu et al., 2012). Generally, perceptual performance is much better for dynamic patterns than static patterns (see Kappers & Plaisier, 2022, for an overview). Therefore, we chose a measure for vibrotactile spatial acuity related to the perception of the direction of sequentially presented vibrations in the current study.

## Method

### Participants

A total of 13 adults (seven female) participated in this experiment. Their ages ranged from 18 to 31 years. Twelve participants were self-reported right-handed, and one was left-handed. The participants were naive as to the purpose of the experiment and they received a financial compensation of 15 euros for their participation. All participants gave written informed consent prior to the experiment. The experiment was approved by the ethical review board of the Human Technology Interaction Group at Eindhoven University of Technology, The Netherlands. We determined the sample size based on an a priori power analysis using $G^{*}$ Power 3 (Faul et al., 2007). We estimated an effect size of f=0.51 for orientation based on a previous study (Kappers et al., 2020) and aimed for a power of 0.9 using an F -test (repeated measures analysis of variance). This resulted in a sample size estimation of 11. Note that due to missing data, two more participants were added resulting in a total of 13 participants. The missing data are also the reason that the final analysis was performed using a linear mixed model (LMM).

### Set-up and Design

**Figure 1.**
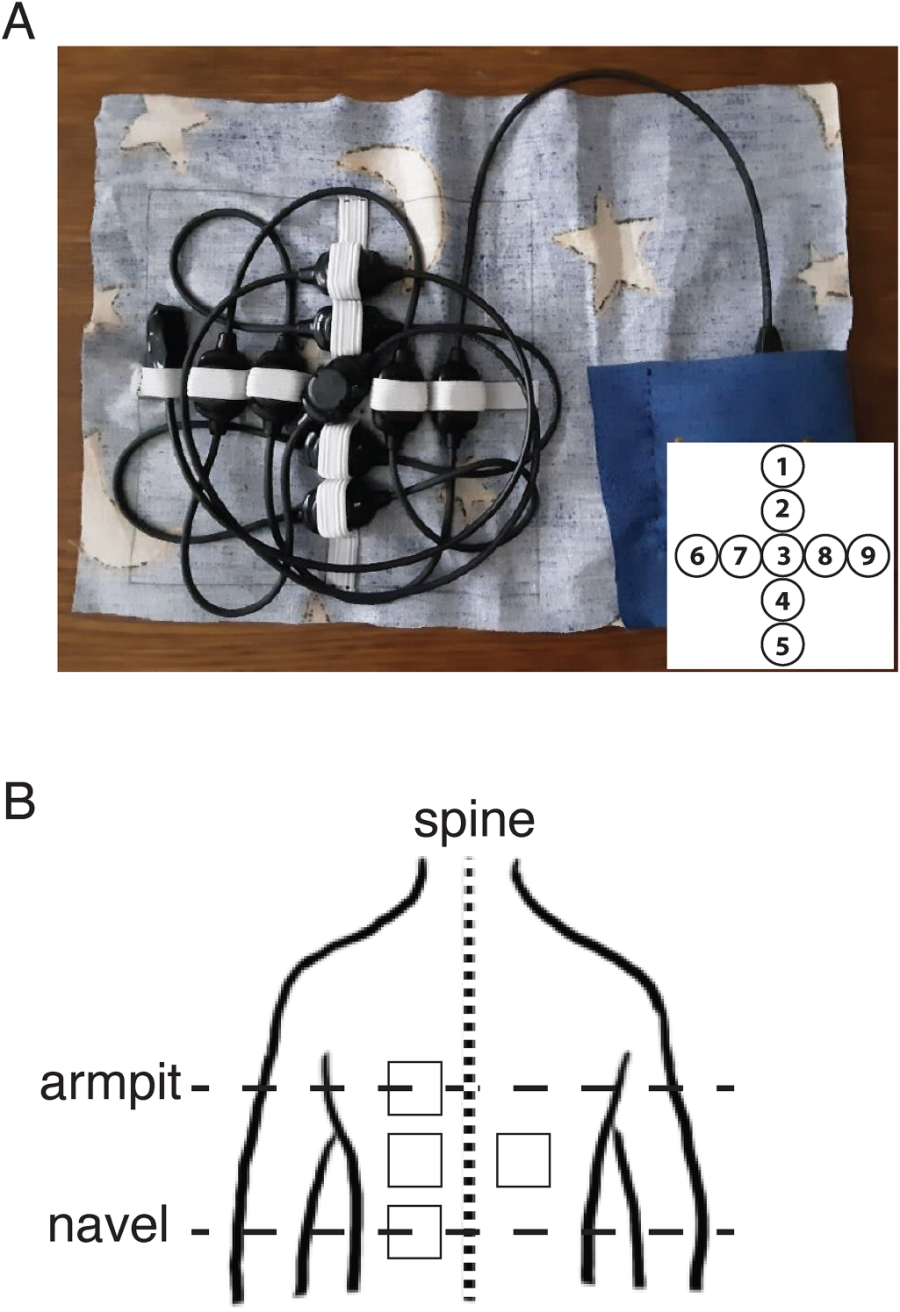
The setup. (A) Picture of the vibrotactile display. The tactors were attached to a piece of fabric using elastic bands to keep them in a cross-like position. The control unit was placed inside a pocket. The inset shows the tactor IDs. (B) Schematic representation of the four back areas that were tested. The squares indicate the back areas of 8 × 8 cm covered by the centre-to-centre distances of the outer tactors. Depending on the size of the back these back areas might slightly overlap.

The device that was used during the experiment was the Tactor string made by Elitac Wearables as a development kit. This kit consisted of nine round tactors that had been overmoulded with thermoplastic polyamide. The tactors consisted of eccentric rotating mass vibration motors (type 312-101, Precision Microdrives). These were coin-style motors which are often incorporated in wearables because they are strong and thus easy to perceive. Additionally, their shape is flat. Each tactor was coin-shaped (diameter 12 mm, height 3.4 mm) and controlled via a TI DRV2604 driver chip (Texas Instruments). This hardware set was controlled via a laptop. The vibration motors were placed in the form of a cross with a horizontal and vertical distance of 8 cm between the centres of the outer tactors. The minimal centre-to-centre distance without the tactors, including the plastic casing, touching each other was about 2 cm. Therefore, a centre-to-centre spacing of 2 cm was used. The tactors were kept in place using a piece of fabric fitted with a small elastic band (see Figure 1A). An elastic textile band was wrapped around the torso to press the tactors onto the participant’s back. The vibration motors were set to the highest setting (15; 100% pulse width modulation) that the device allowed resulting in a frequency of about 150 Hz. This frequency is well within the range of perceivable frequencies (Bolanowski & Zwislocki, 1984) and pilot tests confirmed that the vibrations were clearly perceivable. The amplitude was estimated to be $55.5\pm 9.5\;{m/s}^{2}$ when using standard Elitac Science Suit fastening. Note that the value of the amplitude is quite sensitive to variations in the fastening of the vibration motors and will have varied every time the display was refastened. To accommodate for differences in horizontal curvature near the spine a piece of fabric (wool) was positioned in between the elastic band and the tactors so that the tactors were pressed onto the back to minimise the chances of having uneven pressure against the body. The inset in Figure 1A shows the tactor IDs and Table 1 shows which tactors were used for each presented distance based on these IDs.

**Table 1.** Overview of which tactors were switched on for each distance and each direction.

Distance (cm)	Tactor IDs	Direction
− 2	3 to 7	Left
− 4	8 to 7	Left
− 6	8 to 6	Left
− 8	9 to 6	Left
2	3 to 8	Right
4	7 to 8	Right
6	7 to 9	Right
8	6 to 9	Right
− 2	3 to 4	Down
− 4	2 to 4	Down
− 6	2 to 5	Down
− 8	1 to 5	Down
2	3 to 2	Up
4	4 to 2	Up
6	4 to 1	Up
8	5 to 1	Up

During the experiments, the tactors were always placed such that the centre of the tactor closest to the spine was positioned at 4 cm from the spine meaning that the centre tactor was at 8 cm from the spine. Using the centre point of the centre tactor as a reference the four back areas were defined by the placement of the centre tactor as follows:

- Upper left area: 8 cm left of the spine and at the height of the armpit.
- Middle left area: 8 cm left of the spine and in the middle between the height of the armpit and the navel.
- Middle right area: 8 cm right of the spine and in the middle between the height of the armpit and the navel.
- Lower left area: 8 cm left of the spine and at the height of the navel.

Figure 1B shows a schematic representation of the tested back areas. The experimenter made sure that the vibrotactile display was positioned and fastened correctly. For each back area, the vibrotactile display had to be repositioned. Since we did not have a hypothesis about differences in tactile acuity on the left and right side of the body midline, we chose to measure only the middle back area on both sides of the spine to reduce the data collection time and burden on the participant. The choice to measure three areas on the left side of the spine and only one on the right was arbitrary.

### Procedure

Participants were asked to wear thin clothing (such as a T-shirt) when taking part in the experiment. After placing the vibrotactile display in the first back area, the elastic band was wrapped around the participant’s body horizontally and fastened with Velcro to keep the vibrotactile display in place. Participants wore headphones playing white noise to draw out the sounds from the vibrotactile display. This did not eliminate sound completely as this is also transferred via bone conduction. The sound did not provide any cues with respect to the task, but could be distracting which is why the participants wore the headphones. The experimenter stayed in the room throughout the experiment. The experiments were conducted in a quiet room with only an experimenter and a participant present. Participants sat in front of a computer on a stool or on a chair without touching the backrest.

Before starting a block of trials for a certain back area all tactors vibrated once in sequence and participants were asked to indicate whether they could clearly perceive each vibration. A trial consisted of two tactors sequentially switching on for 500 ms with a break of 500 ms in between. Participants entered the direction in which they perceived the two sequential vibrations via a graphical user interface that displayed arrows (either left and right or up and down) for the participant to click on. Participants were only given the option to answer in the specific orientation of the current condition (i.e. horizontal or vertical). This means that they were not able to answer that they perceived vibrations in the upward or downward direction when the orientation was horizontal, for instance. When the orientation changed, the participants were notified through a pop-up message. Since this was a 2 alternative forced choice design participants needed to be notified in which orientation they were going to be tested.

For each back area, sequential vibrations were presented in the vertical and horizontal orientations in a blocked fashion. Participants performed two blocks in one orientation followed by two blocks in the other orientation. Orientation kept alternating every two blocks throughout the experiment. The distances between sequential vibrations could be 2, 4, 6 or 8 cm and each distance was presented in the direction up or down (vertical orientation) and left or right (horizontal orientation, see Table 1). Each of the 16 distance and direction combinations was repeated 16 times so participants performed 256 trials per back area in blocked random order. The order of the back areas was semi-randomised across participants such that the order was as close to counter-balanced as possible. This means that half of the participants started in the horizontal orientation and the other half in the vertical orientation.

After 256 trials, a popup notified the participant that they should notify the experimenter that they were done with that back area. When the four areas were finished the experiment was completed. Participants could take a short break after each back area. The entire experiment, including measurements and attachment of the device, had a duration of about 90 min per participant.

### Analysis

We defined perceptual precision using the just noticeable difference (JND) and perceptual biases with the point of subjective equality (PSE). These were determined for each back area and each orientation for all participants individually by fitting psychometric functions. This means that eight psychometric curves were fitted per participant. The percentage of answers indicating to the right was calculated for the horizontal trials and the percentage of answers indicating upwards was calculated for the vertical condition for each participant individually. The data were fitted with a cumulative Gaussian using a generalised linear model with a Probit (i.e. a linearised cumulative Gaussian distribution) link-function (Knoblauch & Maloney, 2012). The PSE was defined as the 50% correct point and the JND as the absolute difference between the 50% and the 84% correct points. If the fit of the psychometric function of a participant did not converge, or if the JND was larger than three times the range of stimuli we omitted the PSE and JND for that condition (i.e. back area and orientation combination). There was one participant for whom two JNDs were larger than three times the stimulus range, lower left vertical and middle left vertical, and thus the PSEs and JNDs for these two conditions were omitted. For a second participant, the upper left vertical condition was omitted for the same reason. Finally, there was a participant for whom four psychometric functions didn’t converge (middle left horizontal, middle left vertical, upper left vertical, and middle right vertical) and one for which the JND was larger than three times the stimulus range (lower left vertical). Since this was more than half of all psychometric functions and all vertical conditions for this participant, we decided to omit the full data set for this participant. Because we used an LMM, see below, to compare the JNDs we could omit these back area and orientation combinations for the first two participants instead of having to omit the full data sets of these participants.

**Figure 2.**
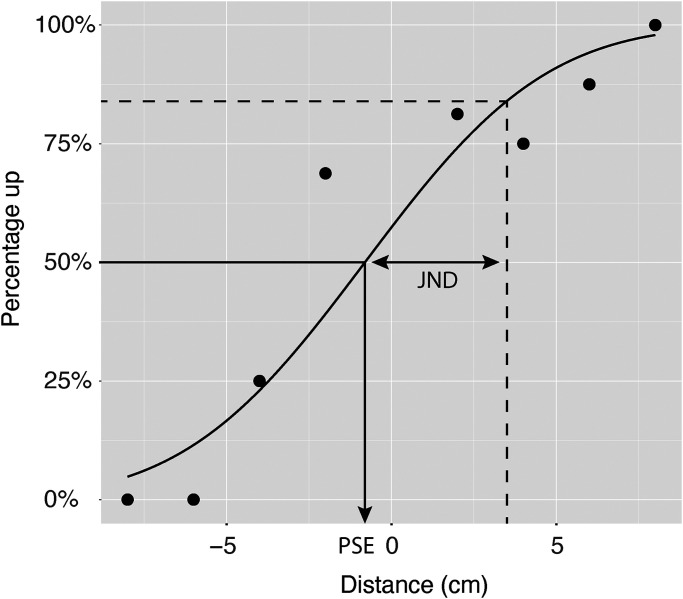
A representative example of a fitted psychometric curve in the middle left back area for the vertical orientation for a single participant. The data points indicate the proportion of “up” responses. The arrows indicate the PSE and the JND. *Note*. PSE = point of subjective equality; JND =  just noticeable difference.

Figure 2 shows a representative example of the fitted psychometric curve for a participant for the vertical orientation in the middle left back area. Negative distances mean that the second tactor was located lower than the first tactor. Similarly, in the horizontal condition, negative distances were defined as the second tactor being located to the left of the first tactor.

After fitting the psychometric functions, the JNDs were analysed by fitting an LMM using the lme4 package in R. This was followed by a deviance test (Wald’s chi-square test) to test for effects of the back area and orientation. The only important aspect about the PSEs in this case is whether there were significant response biases. To test for response bias, the PSEs for each back area and orientation were compared against zero with a two-tailed t -test. A significant difference from zero indicates that there was perceptual bias. Bonferroni correction was applied for multiple testing and the reported p -values are the corrected values.

## Results

**Figure 3.**
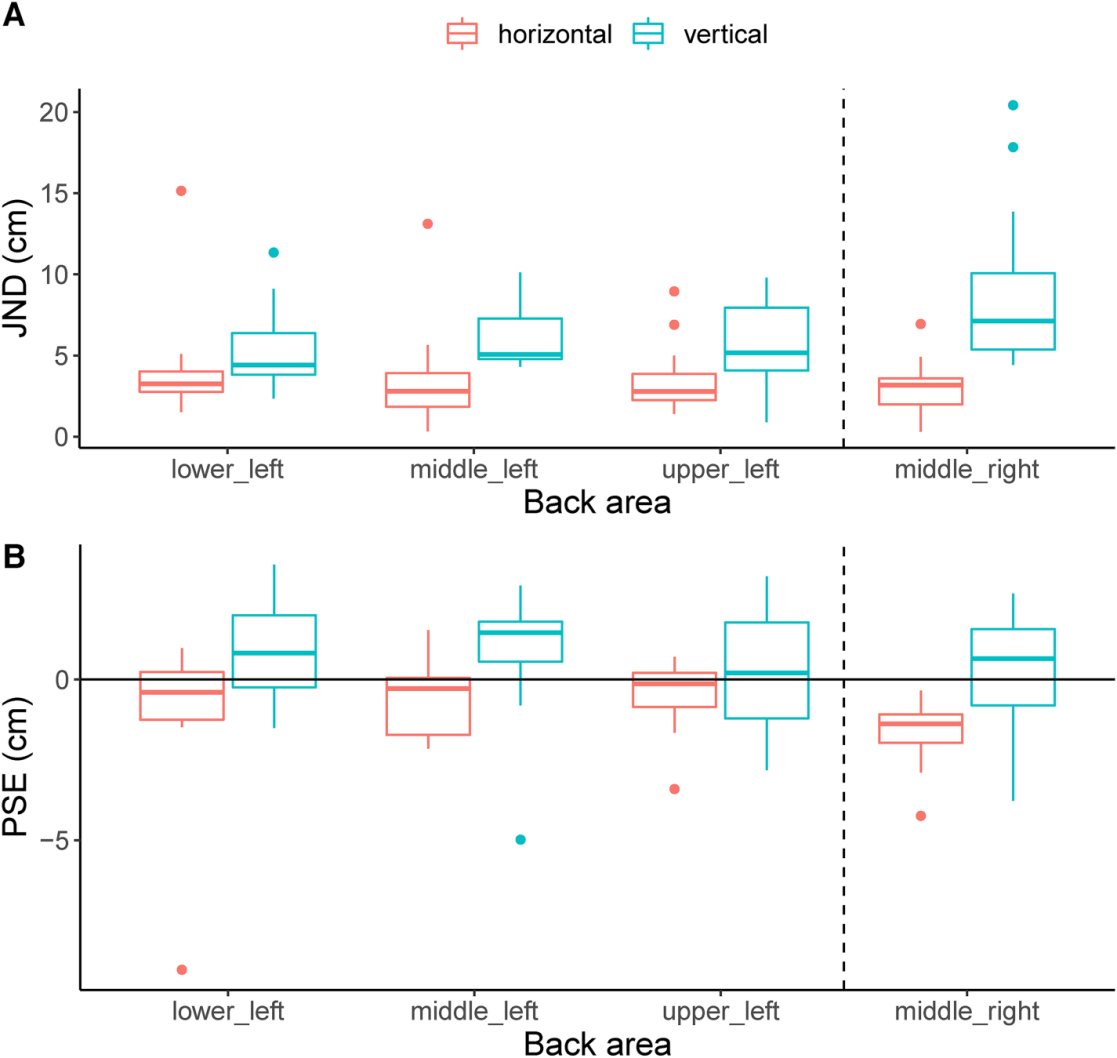
Boxplots of the results for the different back areas and the two orientations. The thick horizontal line indicates the median, the box indicates the 25% to 75% interval and the whiskers indicate the total range without outliers. Dots indicate outliers. The JNDs are shown in (A) and the PSEs in (B). *Note*. JND =  just noticeable difference; PSE = point of subjective equality.

Figure 3A shows a box plot of the JNDs obtained from the fitted psychometric curves for each back area and for both orientations. The vertical dashed line divides the body areas on the left side of the spine from the one on the right side of the spine. It can be seen that the JNDs for the vertical orientation were larger than for the horizontal orientation for all back areas. An LMM was fitted with the JND as a dependent variable and orientation and back area as independent variables. The function was defined in R as follows: JND∼backarea+orientation+backarea:orientation+(1|pp) . A subsequent deviance test showed a significant effect of orientation ( χ(1)=50.1,p<.0001 ), but not of back area ( χ(3)=5.3,p=.15 ) and an interaction between back area and orientation ( χ(3)=13.8,p=.003 ). It can be seen in Figure 3A that the median JNDs were larger in the vertical orientation than in the horizontal orientation for all areas. For some back areas this difference appears to be larger than for others, hence the significant interaction. Post-hoc tests comparing the horizontal and vertical JNDs for each back area showed a significant difference between the middle left area ( p=.001 ) and the middle right area ( p<.0001 ). For the upper left ( p=.07 ) and lower left areas ( p=.07 ), the difference between horizontal and vertical was not significant. Bonferroni–Holmes correction was applied to all reported p -values in the post-hoc analysis. The median JND overall back areas was 3.1±0.8 cm (median ± interquartile range) for the horizontal orientation and 6.6±3.8 cm for the vertical orientation. So spatial acuity differs by roughly a factor of two between the two orientations.

The PSEs are shown in Figure 3B. It can be seen that these were relatively close to zero, which indicates that there were no systematic biases. The only interesting aspect with respect to the PSEs is whether any of these deviate significantly from zero since this indicates a bias. The PSEs were compared to zero using a series of eight two-sided t -tests to determine whether there were perceptual biases for each back area and orientation combination. Only the t -test for the middle right back area in the horizontal orientation showed a significant effect ( t(11)=−5.6,p=.001 , Bonferroni corrected value). This PSE was negative ( − 1.6 cm) indicating a general shift of the psychometric curve towards the left. There was no significant PSE shift in any of the other combinations of back area and orientation. This was not due to the application of the quite conservative Bonferroni correction as the other tests were not significant without Bonferroni correction either.

## Discussion

Our results showed an effect of orientation, but not of the back area. The JNDs were larger in the vertical orientation than in the horizontal orientation by roughly a factor of two. This indicates lower vibrotactile spatial acuity in the vertical orientation. This anisotropy confirms and extends earlier results by Hoffmann et al. (2018) that vibrotactile spatial acuity on the back is higher in the horizontal than in the vertical orientation. It is also consistent with Weber’s findings that tactile spatial acuity is generally higher across the body width (i.e. transverse) than along the body length (i.e. longitudinal) for pressure stimuli (Weber et al., 1996; Ross, 1999). More recently, tactile spatial acuity was shown to be higher in the transverse than longitudinal direction on the hand, wrist and forearm (Cody et al., 2008). Our results for vibrotactile spatial acuity on the back also show that acuity was higher in the transverse direction than in the longitudinal direction. This trend was clearly visible in the data for all back areas, however, the significant interaction between back areas and orientation indicates that for some back areas, the difference might be larger than for others. From the back area and orientation combinations that had to be excluded from the analysis due to the fit not converging it becomes clear that vertical conditions produced JNDs that were far outside of the stimulus range more often than horizontal conditions. This is a clear indication that JNDs were larger in that orientation and that for some participants the JND fell outside the range of stimuli. Overall, our results show that when designing a vibrotactile device for the back it must be kept in mind that there are relatively large differences in spatial acuity depending on the orientation, but not much variability (if any) over different areas of the back.

One important difference between pressure stimuli and vibration is that vibrations can travel through the skin and underlying tissue. This wave propagation differs per body part which can also impact localisation of the vibration (Sofia & Jones, 2013). This wave propagation can differ also depending on the type of vibration motor and is not necessarily the same for the horizontal and vertical directions. So this may be a reason for the differences in JND in the horizontal and vertical directions. This explanation is, however, unlikely given that differences in spatial acuity in the transverse and longitudinal directions have also been reported using pressure stimuli (Weber et al., 1996; Ross, 1999).

We did not find any significant differences for the JNDs across the different back areas. For variations in tactile spatial acuity across the body some general observations have been made. First, more distal parts of a limb generally have a higher tactile spatial acuity (Weinstein, 1968; Weber et al., 1996). In addition to this, Weber found that tactile localisation (i.e. acuteness) is better near a joint, also referred to as an anchor point (Weber et al., 1996). More recently, this has been shown to be the case for localisation on the arm (Cholewiak & Collins, 2003) and the abdomen (Cholewiak et al., 2004). It has been suggested that the spine can be considered an anchor point (Hoffmann et al., 2018). Vibrotactile spatial acuity has been suggested to vary with the distance to the spine. Van Erp has previously reported higher vibrotactile spatial acuity near the spine and navel than in other locations on the trunk (van Erp, 2005). Our results show no systematic variations in spatial acuity across different back areas, which is consistent with both of these observations. The tested back areas all had the same distance to the spine and were equally distal with respect to the body midline.

The analysis of the PSEs showed only a significant difference from zero for the horizontal orientation condition in the middle right back area. This indicates that in this condition the whole psychometric curve is shifted to the left. This means that participants responded more often “right” than “left” when the second vibration was presented somewhat to the left of the first vibration. This bias was not expected and since it was only significant for one of the PSEs it is difficult to interpret making the meaning of this result limited. A recent study found that localisation of pressure stimuli shows a bias towards the periphery (Pratt et al., 2024). However, it is too early to conclude that our results are consistent with these findings, as discrimination thresholds and the PSEs are not necessarily affected by localisation errors and vibration stimuli are quite different from pressure stimuli.

For the design of vibrotactile displays for the back, our results mean that the distance between tactors can be the same for the back areas that were included in this study. However, tactor density in the vertical and horizontal orientations can be different. Our results show a higher vibrotactile spatial acuity (i.e. smaller JND) for the horizontal orientation than the vertical orientation and therefore tactor density in the vertical orientation can be lower than in the horizontal orientation. By using an anisotropic tactor density the number of tactors can be minimised. This can lower production costs and simplify the control of the actuators without sacrificing user experience.

As discussed above tactile spatial acuity varies over different body areas and for different orientations on a body part (Weber et al., 1996; Cody et al., 2008; Weinstein, 1968). There also exists an effect known as Weber’s illusion (Weber et al., 1996). This is the effect that a distance between two points of tactile stimulation feels larger on a body part with high acuity than on a body part with low acuity. In the above, we reported an anisotropy in acuity for transverse and longitudinal directions and thus we may expect also a difference in perceived distance between these two directions. Indeed, using pressure stimuli this difference in distance perception between transverse and longitudinal directions has been shown to exist for many body parts (e.g. Longo, 2020; Fiori & Longo, 2018; Longo & Haggard, 2011; Nicula & Longo, 2021; Green, 1982; Stone et al., 2018). Based on the results of the current study for vibrotactile spatial acuity it would thus be expected that the distance between two vibrations in the horizontal orientation on the back would feel larger than the distance between two vibrations in the vertical orientation on the back. In a previous study, however, we found the opposite effect as participants judged distances between two vibrations in the vertical orientation on the back to be larger than in the horizontal orientation (Plaisier et al., 2020). This result has since been confirmed by other researchers using pressure stimuli (Nicula & Longo, 2021). Distance to the spine might play a role here, since the vibrations in the study on vibrotactile distance perception were located closer to the spine (3 to 4 cm) than in the current study (8 cm). An alternative explanation could be that the presented distances in the vertical orientation on the back were parallel to the spine and that the spine could act as a reference. Whether this is indeed the case needs to be investigated further.

### Conclusion

Previously, some of the authors have proposed design guidelines for vibrotactile displays based on previous studies on vibrotactile spatial acuity, distance and orientation perception on the back (Kappers & Plaisier, 2022). In those guidelines, we suggested a tactor grid which is more elongated in the vertical orientation to counteract perceptual deformations when tracing shapes. The current findings also provide a reason to space tactors closer together in the horizontal direction than in vertical orientation. The JNDs were roughly twice as large in the vertical orientation than in the horizontal orientation. This is a considerable difference and therefore a lower tactor density in the vertical orientation than in the horizontal orientation can be used to minimise the number of tactors.
